# Effects of Sintering Temperature Variation on Synthesis of Glass-Ceramic Phosphor Using Rice Husk Ash as Silica Source

**DOI:** 10.3390/ma13235413

**Published:** 2020-11-28

**Authors:** Rabiatul Adawiyah Abdul Wahab, Mohd Hafiz Mohd Zaid, Sidek Hj. Ab Aziz, Khamirul Amin Matori, Yap Wing Fen, Yazid Yaakob

**Affiliations:** 1 Department of Physics, Faculty of Science, Universiti Putra Malaysia, Serdang 43400, Malaysia; rabiatul6341@uitm.edu.my (R.A.A.W.); sidek@upm.edu.my (S.H.A.A.); khamirul@upm.edu.my (K.A.M.); yapwingfen@upm.edu.my (Y.W.F.); yazidakob@upm.edu.my (Y.Y.); 2 Faculty of Applied Sciences, Perak Branch Tapah Campus, Universiti Teknologi MARA, Tapah Road, Perak 35400, Malaysia; 3 Materials Synthesis and Characterization Laboratory, Institute of Advanced Technology, Universiti Putra Malaysia, Serdang 43400, Malaysia

**Keywords:** rice husk ash, zinc-boro-silicate, willemite, structural, luminescence

## Abstract

In this study, the authors attempted to propose the very first study on fabrication and characterization of zinc-boro-silicate (ZBS) glass-ceramics derived from the ternary zinc-boro-silicate (ZnO)_0.65_(B_2_O_3_)_0.15_(RHA)_0.2_ glass system through a conventional melt-quenching method by incorporating rice husk ash (RHA) as the silica (SiO_2_) source, followed by a sintering process. Optimization of sintering condition has densified the sintered samples while embedded beta willemite (β-Zn_2_SiO_4_) and alpha willemite (*α*-Zn_2_SiO_4_) were proven in X-ray diffraction (XRD) analysis. Field emission scanning electron microscopy (FESEM) has shown the distribution of willemite crystals in rhombohedral shape crystals and successfully form closely-packed grains due to intense crystallization. The photoluminescence (PL) spectra of all sintered ZBS glasses presented various emission peaks at 425, 463, 487, 531, and 643 nm corresponded to violet, blue, green, and red emission, respectively. The correlation between the densification, phase transformation, microstructure, and photoluminescence of Zn_2_SiO_4_ glass-ceramic phosphor is discussed in detail.

## 1. Introduction

Rice husk ash (RHA) is one of the significant agriculture by-products, produced yearly, about 0.5–0.6 million tons from the annual paddy cultivation, particularly in Malaysia. This silicate-based material is often abandoned in the landfill or opened burnt to be eradicated shortly after the harvest season due to its least commercial value. Thus, this has become a problematic issue for the rice-producing countries in managing the disposal of its industrial waste. RHA slowly breaks down because of its high lignocellulosic content that delayed the degradation process and the high carbon (C) and silica (SiO_2_) content that makes it hardly degrade over time [1]. Thus, researchers have been actively studying the extraction SiO_2_ from RHA employing conventional pyrolysis of rice husk (RH) to assist rice-producing countries facing incompetent RH disposal management.

RHA is low in density (350–850 kg/m^3^) and high in porosity, which makes it best suited as a potential thermal insulative precursor [2]. RHA’s incorporation has been studied in the vitrification of glasses, ceramics, and glass-ceramics over the centuries [3,4,5]. The highly reactive SiO_2_ content in RHA is suitable for SiO_2_ replacement as a host matrix over the high purity SiO_2_ powder. Heat-treated RHA may content diphasic crystalline phases such as cristobalite and tridymite, which is highly temperature-dependent. Moreover, the highly reactive SiO_2_ content may make RHA the potential waste-based precursor in making geopolymer and alkali-activated materials. These materials were successfully applied in cement, concrete, and pavement [6,7,8]. Thus, it is undoubtedly a big hit in the construction industry, which incessantly useful in creating a greener environment.

Reinforcement of RHA in glasses successfully fabricated various enhanced host glass matrix of silicate-based glass such as aluminosilicate glass, borosilicate glass, calcium silicate glass, and bio-active glass [9,10,11,12]. Silicate glasses with basic structural unit of a tetrahedron of SiO_4_ are linked at each corner where each oxygen is bonded to two silicon ion, Si^2+^, with a very well-defined geometry. The co-existence of alkaline oxide in RHA acts as a network modifier that facilitates the melting process, which reduces the appearance of bubbles entrapped in the glass bulk [13]. This glass’s high melting point at 1723 °C makes it thermally resistant to thermal shock and suites the best for technical glass applications range from optical lenses, windowpane, and optical fiber. Whereas, ZnO with a high refractive index ~2.0 [14] itself can’t form a glass, but rather it needs to be paired with other intermediate glass host matrix, which can make it act as either former or network modifier. The presence of zinc ion, Zn^2+^ in zinc silicate (ZnO-SiO_2_) binary glass system contributes to the glass matrix’s low softening point. Due to its structural behaviors within the glass network, the ZnO-containing glass system improves optical properties and luminescence properties ascribed to the wide bandgap (~3.37 eV) at room temperature [15]. The increment of Zn^2+^ has increased the transparency of the ZnO-SiO_2_ glass due to the increment of non-bridging oxygen that is affected by the breakage of Si-O-Si bonds [16]. Up-to-date, intensive fabrication of glass-ceramic over glass is directly related to modifying its physical improvement, mechanical strength, heat resistance, and chemical stability [17]. Thus, the vital requirement is the amount of embedded crystal in the glass matrix utilizing a controlled sintering environment.

Over the years, the fabrication of zinc silicate (Zn_2_SiO_4_)-based glass-ceramics has attracted massive attention for its wide bandgap as it has promising optical transparency and significant fluorescence characteristics. It is manufactured attentively for laser diodes, optical communications, electronic storage, technical glasses, solar absorptive coating, and optical-electronic devices [18,19,20,21,22,23,24]. The processing techniques are covered from conventional sol-gel, thermal treatment, solution combustion, a hydrothermal and solvothermal method [25,26,27,28]. These few decades seem stringent as researchers are diligently looking into the simplicity of solid-state technique in producing Zn_2_SiO_4_. Instead, they are struggling with melting the admixture precursors powder to form the ZnO-SiO_2_ host glass. Previous studies showed a high temperature required to melt the ZnO-SiO_2_ binary glass system [29,30,31,32]. As for the ZnO-SiO_2_ admixture, the melting environment lingers around 1400–1500 °C to reach complete molten [33,34,35]. The addition of boron oxide (B_2_O_3_) into the glass matrix network is considered a valuable finding, which resulted in the glass system having improved physical and chemical properties. A comparative study on the zinc-boro-silicate (ZnO-B_2_O_3_-SiO_2_) has been done thoroughly; thus, variation in ZnO/B_2_O_3_ ratio has guided the actual proportion to produce precipitated crystals which is later proven that with the B_2_O_3_ additive, it can solve the crucial issue of lowering the melting point compared to ZnO-SiO_2_ [36,37,38]. The incorporation of ZnO-B_2_O_3_-SiO_2_ (ZBS) as a ternary glass system has developed willemite (Zn_2_SiO_4_) crystals through the sintering process. To date, this host glass system has been beneficially studied as the parent glass for rare-earth dopants and successfully produced red, green, yellow, and blue phosphors, which can act as a promising semiconductor material in electronic devices [39].

As far as is known, limited studies were reported on the fabrication of the ternary zinc-boro-silicate (ZBS) system based on ZnO-B_2_O_3_-RHA composites. Hence, the effect of sintering temperature variation on the structural and optical performance of the ZBS glass were described intricately. The properties of RHA were explicitly examined through chemical and structural studies. The physical and structural properties of the sintered ZBS glass were investigated. Other than that, the luminescence properties of the ZBS glass-ceramics were evaluated and discussed. Hence, the focus of this work is to synthesize ZBS composite by incorporating RHA as the reactive SiO_2_ source for the host matrix and also to perform an investigation on structural and luminescence properties subjected to sintering temperature by studying the development in physical changes, compositional structures, and bonding, and optical illumination. Therefore, the ZnO-B_2_O_3_-RHA composite will be the host matrix for the fabrication of ZBS glass-ceramics to develop potent phosphor material in the optoelectronic industry.

## 2. Materials and Methods

### 2.1. Preparation and Synthesis of Silica (SiO_2_) from Rice Husk

**Figure 1:**
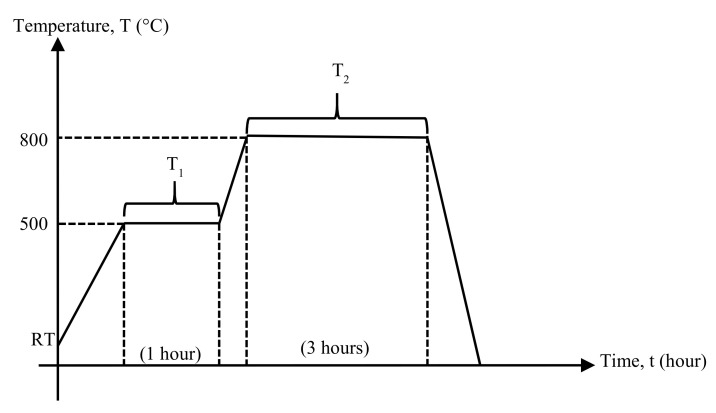
The double stage combustion process of burning raw rice husk.

The raw rice husk (RH) was supplied by the rice paddy mill company, BERNAS; Kuala Selangor, Malaysia. It was then cleaned with tap water to remove the mud, dirt, and impurities. Then, further rinsing was done three times using distilled water to eliminate the residual dirt and impurities as the final cleaning steps. Next, the RH was dried in an electric oven at 120 °C for 24 h to reach complete dryness, followed by the dual combustion stage (DCS) process (refer Figure 1). An amount of 10 g dried raw RH was placed in a 150 mL cylindrical alumina crucible. The heat treatment process to acquire rice husk ash (RHA) as the silica (SiO_2_) active based materials started from room temperature (RT) to 500 °C for 47.3 min. Followed by the first stage, T_1_ required heating temperature around 500 °C for 1 h and the second stage, T_2_ with the heating temperature at 800 °C for 3 h under the heating rate of 10 °C/min. Later, the cooling process adhered to the heating rate set but without the cooling agent’s assistance, it may take longer than 77.3 min to reach RT. Afterward, laboratory agate mortar and pestle were used to grind RHA into finer powder for further study, mainly chemical and structural properties. The chemical compositions were acquired from X-ray fluorescence (XRF) spectroscopy (Fluorescence X-ray Spectrometer EDX-720/800HS/900HS, SHIMADZU, Tokyo, Japan), whereas the structural properties were analyzed by X-ray diffraction (XRD) spectroscopy (PW3040/60 model, PHILIPS, Kanagawa, Japan).

### 2.2. Synthesis of Zinc-Boro-Silicate (ZBS) Glass-Ceramic

The ternary system of ZBS host glass was prepared based on the empirical composition of 65ZnO-15B_2_O_3_-20RHA by using precursors of rice husk ash (RHA), zinc oxide (ZnO, 99.9% purity, brand Sigma Aldrich, St. Louis, MO, USA), and boron oxide (B_2_O_3_, 99.9% purity, brand Sigma Aldrich, St. Louis, MO, USA). The mixture was then dry milled in high-quality laboratory agate mortar and pestle to ensure a well-combined mixture was produced. Then, 40 g of the mixture was placed into a 1000 mL alumina crucible to be subjected to a melting procedure at 1350 °C for 2 h in order to be completely turned into a glass molten. The molten was then poured down into a tap water bath which is set at room temperature for a quenching step, resulting in glass frits fully submerged in the water bath. The collected glass frits weighed about 32 g, and then they were let to dry overnight in ambient condition. Later, the glass frits transformed into glass powder after being crushed, ground, and sieved into ≤45 μm size fine particles using an industrial sieve. To form ZBS glass-ceramics, 1 g fine glass powder was premixed with an organic binder, with the addition of polyvinyl alcohol (PVA), then was pressed into compacted disc shape samples with fairly ~13 mm in diameter and thickness of ~3 mm by using a hydraulic pressing machine. Then the green bodies were subjected to a sintering process at 600 °C, 700 °C, 800 °C, and 900 °C in an electrical furnace with a heating rate of 10 °C/min for 2, 4, and 10 h. Each sample was denoted as A, B, C, and D, and 1, 2, and 3, as elaborated in Table 1. The sintered samples were later crushed, grind, and sieved into finer powder characterized by physical, microstructural, and luminescence characteristics.

### 2.3. Characterization of Physical Properties

The sintered samples were transformed into powder beforehand to measure the true density. Afterward, the measurement was carried out using a micromeritics gas pycnometer (AccuPyc II 1340, KROMTEK, Tokyo, Japan) in the medium of helium gas at room temperature. The powder was placed in a cylindrical steel mold of size 1.0 cm^3,^ and the mass of the powder, m_s_, was measured prior. Then, the mold was transferred into the sample chamber of the pycnometer. Boyle’s Law was employed to acquire the true density by the following equations:

P_1_V_1_ = P_2_V_2_ (1)

P_1_ is pressure 1, V_1_ is volume 1, P_2_ is the pressure 2, and V_2_ is the volume 2. P_1_ is the pressure for the sample chamber and P_2_ is the pressure inside the expansion chamber.

(2) VS=VC−VEP1P2 − 1 

where V_S_ is the volume of sample, V_C_ is the volume of the sample cell, V_E_ is the volume of expansion cell. Next, the true density measurement executed by the following equation:

(3) ρT= mSVS

where $\rho_{T}$ is the true density of the sample, $m_{S}$ is the mass of sample and $V_{S}$ the volume of the samples. The measurement was taken 5 times to ensure the high accuracy of the data.

### 2.4. Characterization of Structural and Luminescence Properties

The crystallinity and crystal growth of the sintered samples were confirmed using the XRD measurement (Philips, PW3040/60 model) at 20–80° in the range of 2θ and analyzed utilizing an XRD analysis software, PANalytical X’Pert Highscore Plus. The sintered samples’ topographical features and microstructural properties were studied through field emission scanning electron microscope (FESEM), using NanoSEM 230, FEI NOVA, Hillsboro, OR, USA.

The luminescence emission was identified using a photoluminescence spectrometer (PERKIN ELMER, Waltham, MA, USA), LS 55 model). The sintered samples in powder form were subjected to UV light under the AS ONE handy UV Lamp at room temperature.

## 3. Results and Discussion

### 3.1. Chemical and Structural Characterization of Rice Husk Ash (RHA)

**Figure 2:**
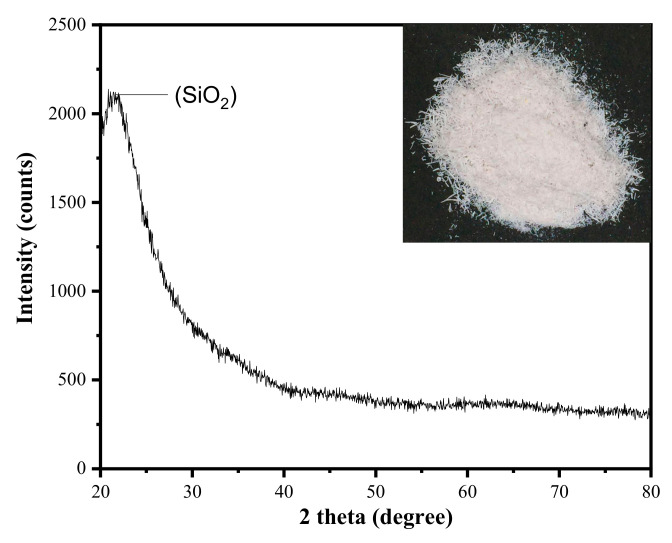
XRD pattern of heat-treated RHA.

The raw rice husk (RH) which was brownish in color, had turned into white RHA (refer Figure 2) after heat-treated through the DCS process (refer Figure 1). The RHA has been further studied on the chemical and structural properties through XRF and XRD spectroscopy. Table 2 confirmed the SiO_2_ content was about 95.6%, with the other co-existing metallic oxides were presumed as impurities, while Figure 2 demonstrated the SiO_2_ structural phase, thus represented the diffraction pattern that can show the amorphous or crystalline nature of the analyzed sample [31].

It also shows the single prominent peak identified at 2θ = 22° with a small-scale shoulder attributed to SiO_2_. The hump represented in the low region mainly indicated the presence of the amorphous nature of SiO_2_. This XRD pattern was consistent with the silicate network co-founded by the latest studies in [16]. In this study, it was crucial to ensure RHA was amorphous as the crystalline precursor’s usage may require a high melting temperature to form the host glass, thus hindering the crystallization of zinc-silicate based glass during the sintering process to develop glass-ceramics [33,34,40].

### 3.2. Characterization of the ZnO-B_2_O_3_-SiO_2_ (ZBS) Glass

**Figure 3:**
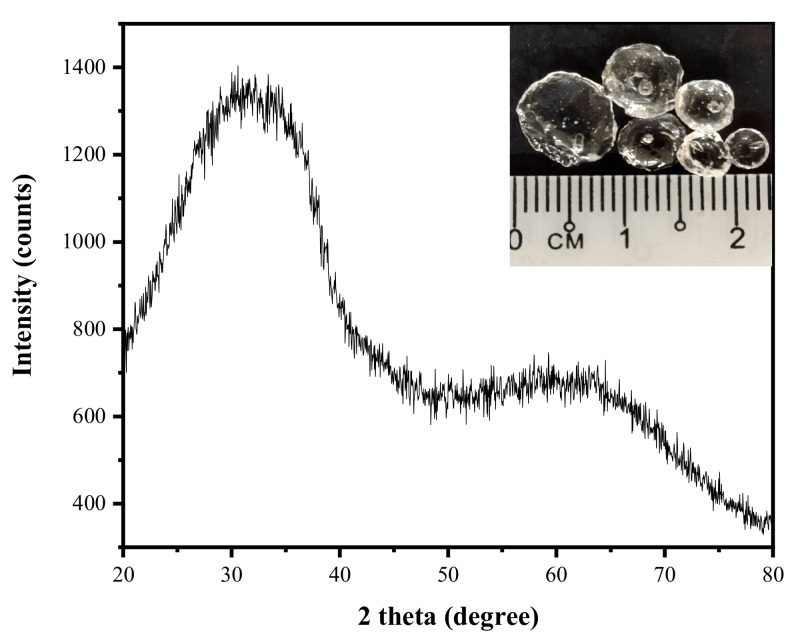
XRD diffraction pattern and the formation of glass frits from the ZnO-B_2_O_3_-SiO_2_ (ZBS) glass.

The phase formation in the ZBS glass was obtained by using an x-ray diffraction (XRD) spectrometer and analyzed by the software patented. Figure 3 below shows the XRD pattern of the ZBS glass formed due to the melt and quench process, thus presented the appearance of a transparent ZBS parent glass in the form of glass frits. The figure was in line with the previous study, which has successfully produced transparent zinc silicate glass. The ternary ZnO-B_2_O_3_-SiO_2_ glass sample was successfully fabricated at 1400 °C and poured into a tap water bath at room temperature (~27 °C). The glass frits appeared transparently clear and were deemed homogenous as the molten was easily poured into water. The broad humped at 32° with no sharp diffraction pattern indicated that no regular atomic arrays have formed, demonstrating no crystalline phase exhibited in the glass frits.

### 3.3. Physical Characterization of the ZnO-B_2_O_3_-SiO_2_ (ZBS) Glass-Ceramics

Physical characteristics of the glass-ceramics have been investigated through true density, which was an effective way to study the structural changes with respect to the heat treatment environment. The sintering process was often treated as the potent factor that drives the deliverance of surface free energy on the bulky samples. Thus, this may relieve the intermolecular bonds and cause grain boundary diffusions, resulted in bulky changes of the green bodies.

**Figure 4:**
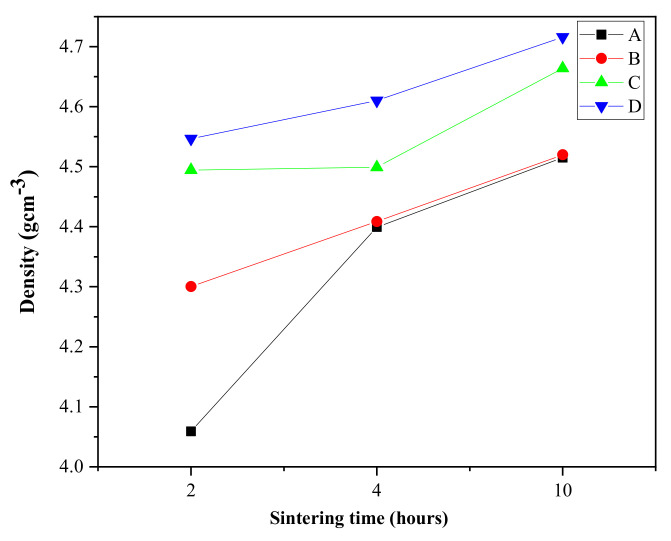
The density of the ZBS glass-ceramics is sintered at various temperatures and holding time.

Figure 4 below shows the pattern of true density of the compacted ZBS sintered at 600–900 °C and hold for 2, 4, and 10 h, respectively. The density, which increased with the sintering time and temperature from 4.059 to 4.716 g/cm^3^, portrayed rapid densification. This pattern significantly represented the actual behavior of atomic diffusion, where diffusivity was increasing proportionally to the temperature applied [41]. Higher temperatures supplied sufficient energy causing the grain boundaries diffusion to happen between the host particles. The crystal growth gave expenses on the smaller grains, thus eliminating voids located inter-grains and increased the atomic structures [42]. Thus, high sintering temperature and time showed structure compaction and resulted in a denser grain packing and total apparent porosity removal.

The difference in true density and bulk density (refer Table 3) were measured for each sintering temperature, varied with holding time of 10 h from 22.09 to 30.92% and it was proven that bulk density was slightly lower than true density because apparently bulk density did not measure the open and close pores.

### 3.4. Structural Characterization of the ZnO-B_2_O_3_-SiO_2_ (ZBS) Glass-Ceramics

**Figure 5:**
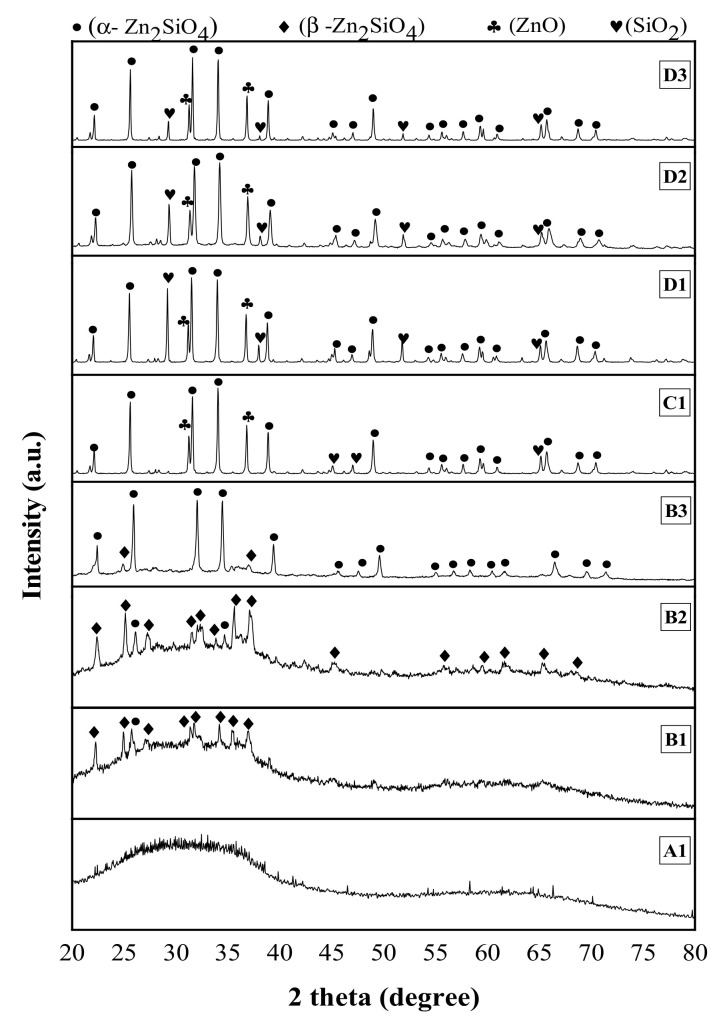
XRD diffraction spectrums of the sintered ZnO-B_2_O_3_-SiO_2_ composite.

**Figure 6:**
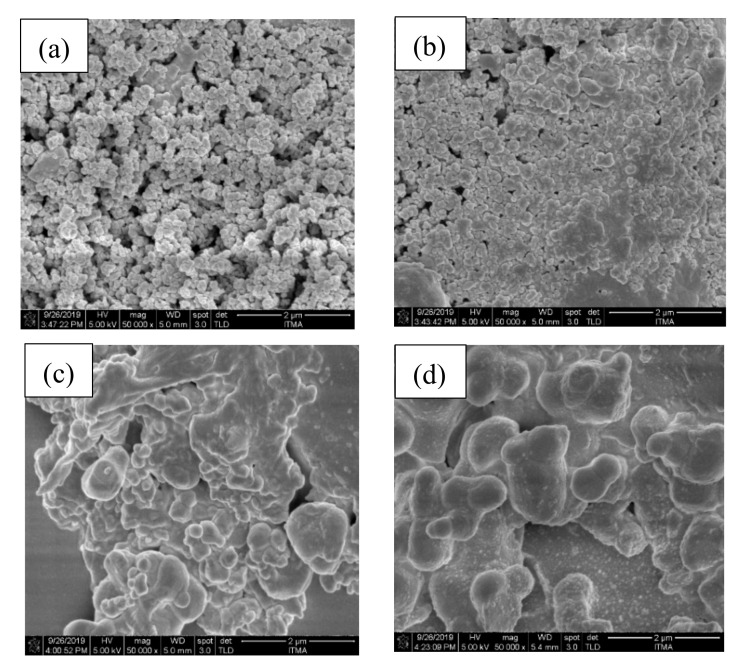
FESEM images of ZBS glass-ceramics samples (**a**) A1, (**b**) B1, (**c**) C1, and (**d**) D1.

**Figure 7:**
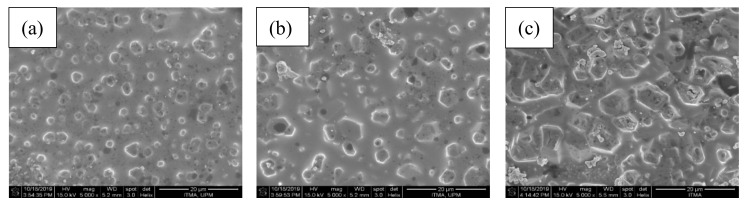
The surface morphological view of the ZBS glass-ceramics samples (**a**) D1, (**b**) D2, and (**c**) D3.

The structural characteristics of the ZBS glass-ceramics that have been sintered at various temperatures and time were characterized through XRD measurement and micrographs images utilizing FESEM, thus discretely presented in Figure 5, Figure 6 and Figure 7. As shown in Figure 5, the XRD spectrum of ZBS glass sintered at the lowest sintering temperature remains in amorphous nature as the corresponding peak is quite low in intensity with a broad shoulder. The pattern was in fully amorphous behavior as it showed the broad halo pattern at around 2θ = 30° as no crystallization process occurred yet. This behavior lasted the same even after compacted ZBS glass was sintered for 4 and 10 h with the same sintering temperature, 600 °C that is not shown here. Anyhow, the glass-ceramic samples sintered for higher temperature, 700 °C, started to form a metastable crystalline phase of *β*-Zn_2_SiO_4_ with JCPDS file No. 19-1479. It can be seen that the formation of *β*-Zn_2_SiO_4_ had been intensified when sintered for a long time, suggesting that sample B2 was in unstable condition [43]. With sufficient activation energy, sample B3 started to form thermodynamically stable crystalline phase of α-Zn_2_SiO_4_ (JCPDS file no. 37-1485) that was prominently appearing at 2θ = 22.19°, 25.66°, 31.65°, 34.12°, 38.94°, 45.14°, 47.07°, 49.03°, 54.39°, 56.14°, 57.70°, 59.63°, 60.97°, 65.73°, 68.77°, and 70.45° corresponding to (3 0 0), (2 2 0), (1 1 3), (4 0 1), (2 2 3), (4 1 3), (5 2 0), (3 3 3), (6 0 3), (5 2 3), (7 1 0), (0 0 6), (6 3 0), (7 1 3), (6 3 3) and (4 6 1).

Only by anatomizing the diffraction spectrums, it was worth noting that the intensity of diffraction peaks of α-Zn_2_SiO_4_ increase and become sharper as the ZBS glass sintered up to 900 °C due to the longer duration of sintering applied. Therefore, the intensity of Zn^2+^ and Si^4+^ ions diffusion increased; hence, the crystals’ growth rate was enhanced. The crystalline ZnO (JCPDS 79-2205) appeared at 2θ = 31.78°, and 36.26°, together with SiO_2_ (JCPDS 75-0638) started to appear at 800 °C in sample C1, slowly disappeared in higher sintering temperature, which indicated the greater diffusion of Zn^2+^ and Si^4+^ ions to form α-Zn_2_SiO_4_. Even after maximum sintering temperature and holding time applied, the peaks of ZnO and SiO_2_ still left, suggesting further sintering at a higher temperature may intensify the α-Zn_2_SiO_4_ crystallization [44].

The changes in the topographical structure of the ZBS glass-ceramics were studied through FESEM. As shown in Figure 6, the FESEM micrographs at a magnification level of 50,000 showed the microstructures of the green bodies sintered at 600–900 °C for 2 h. It can be observed that the micrographs revealed that the sintering process greatly influenced the changes in topographical structure. The lowest sintering temperature, 600 °C, depicted the agglomeration of particles in irregular equiaxed shape. At 700 °C, closely-packed grains of willemite crystals dominated the micrograph, where the particle distribution was irregular. Quite simply, at the higher sintering temperature, particles started to aggregate where two particles merged and formed a neck between each other. Such neck formation led to the sample’s densification, thus producing a more compact structure and grain sizes are generated and consequently lower the porosity.

It can also be observed from the other micrographs at a magnification level of 5000, a well-formed willemite crystal after sintered at 900 °C for 2, 4, and 10 h were shown in Figure 7. The formation of Zn_2_SiO_4_ can confirm this in rhombohedral-like particles that started to show after sintered at 900 °C for 2 h. An increment of holding sintering time would relatively accelerate the growth of the rhombohedral-like particles, which densified the samples over time. The FESEM observation showed that the voids were removed in higher holding time as willemite crystallization intensified [45].

### 3.5. Optical Characterization of the ZnO-B_2_O_3_-SiO_2_ (ZBS) Glass-Ceramics

**Figure 8:**
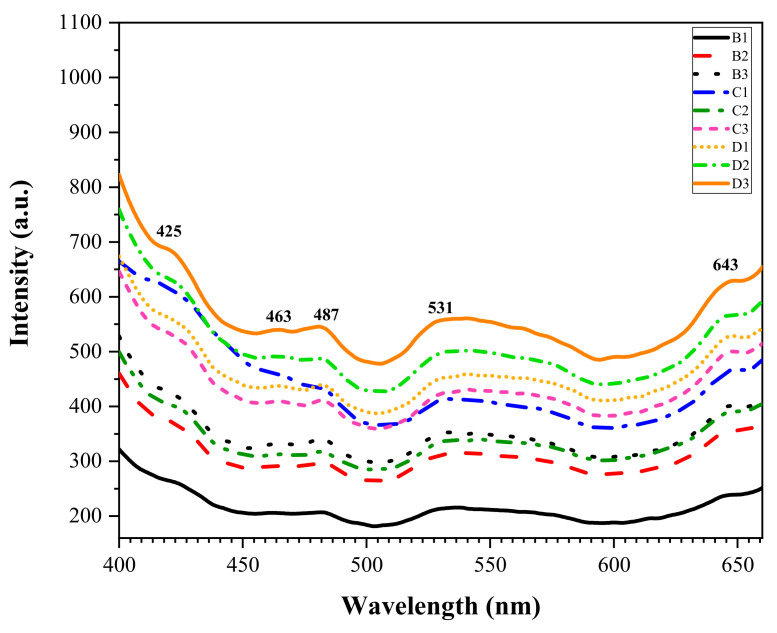
The photoluminescence spectra of ZBS glass-ceramics with excitation at 360 nm.

The luminescence properties were studied by employing a photoluminescence (PL) spectrometer to analyze and generate information on the tested samples’ emission spectra. As can be observed in Figure 8, the emission spectra were recorded in the wavelength range of 400–650 nm, excited at 360 nm, where the previous study inspired this selected excitation wavelength onto the ZBS glass system [46,47]. Throughout the ZBS samples sintered at various temperatures for different holding time, the spectra exhibited five different emission peaks in violet, blue, green, yellow, and red spectrum located at 425, 463, 487,531, and 643 nm, respectively. Prominently, all the observed peaks intensified as the sintering temperature increased with the increment of sintering holding time with no change in the PL peak position.

The highest emission spectra corresponded to the ZBS sample sintered at 900 °C for 10 h, with the most intense peak at 531 nm corresponded to the green spectrum. The Zn^2+^ ion was highly occupied in this sample caused the breakage of oxygen bonds that increased non-bridging oxygens (NBO’s). Therefore, it led to the increment of absorption of the electron, thus making the emission spectra intensified throughout the sample. Besides, the structural defect occurred in ZnO and Zn_2_SiO_4_ due to the zinc vacancy played a crucial part as a deep acceptor that developed the green luminescence. The violet-blue emissions centered a 425 and 463 nm were expected to originate from defect pair contributed by the SiO_2_ matrix in the form of dioxasilyrane and silylene [48]. Meanwhile, the other study provided a significant finding of blue emission pronounced in ZBS samples due to the ZnO crystalline phase [49]. It was rarely found in other zinc silicate-based host matrix as usually, green and yellow were commonly found in general [50,51]. Meanwhile, an intensified red band prominently occurred at 643 nm is due to the structural defect in ZnO that appeared in the form of oxygen vacancy and zinc interstitial [16,40], which was also found earlier in [52].

**Figure 9:**
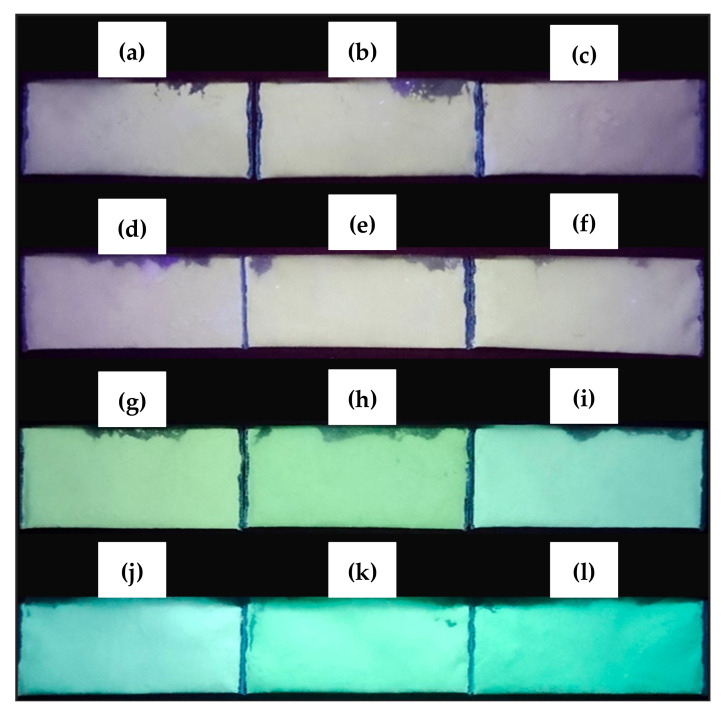
The persistent emission by the ZBS glass-ceramics under the beam of UV light illumination with excitation of 254 nm: (**a**) sample A1, (**b**) sample A2, (**c**) sample A3, (**d**) sample B1, (**e**) sample B2, (**f**) sample B3, (**g**) sample C1, (**h**) sample C2, (**i**) sample C3, (**j**) sample D1, (**k**) sample D2, and (**l**) sample D3.

Figure 9 presents the emission of dual-spectrum by ZBS samples sintered under different temperatures and various holding time. The limitation offered by the available UV lamp source under the low-wavelength region (254 nm) was the closest excitation spectra of ZnO-based photoluminescence in previously studied ZnO-SiO_2_ host system [53]. The ZBS sintered samples have exposed the transition of yellowish to greenish emission, where all the samples prepared were initially off-white. Samples A1–A3 (Figure 9a–c) and B1–B3 (Figure 9d–f) appeared slightly yellowish due to the presence of *β*-Zn_2_SiO_4_ which was responsible for the yellow emission. Whereas the green emission was evident in samples C1–C3 (Figure 9g–i) and D1–D3 (Figure 9j–l) sintered under various holding times indicated the occupancy of α-Zn_2_SiO_4_ [54]. Particularly, samples C appeared in yellowish-green. The higher sintering temperature gave more intensified green emission, such as samples D, due to ZnO stable direct bandgap, which promises more efficient exciton emission at higher temperatures [55].

## 4. Conclusions

In this recent study, zinc borosilicate (ZBS) glass-ceramics were successfully prepared using conventional melt-quench of the ZnO-B_2_O_3_-RHA ternary glass system. The density of the sintered green bodies increased as the sintering temperature increased. Moreover, the increment sintering temperature intensified the formation of willemite crystals from *β*-Zn_2_SiO_4_ to *α*-Zn_2_SiO_4,_ which both started to form at 700 °C but at different holding time. It was supported by the evidence from FESEM micrographs showing the optimized sintering temperature applied fairly grown to the willemite crystal in rhombohedral-like shape and resulted in a closely-packed grain. Finally, PL emission spectra executed violet, blue, green, yellow, and red colors, indicating a good luminescence performance and applied as an optoelectronic device. Therewithal, this study could be useful for the development of rare-earth-doped with ZBS as a potential glass-ceramics which potentially produced white light emission.

## Figures and Tables

**Table 1:** Name of the sample according to the sintering temperature and holding time.

Sample(s)	Sintering Temperature (°C)	Holding Time (Hours)
A1	600	2
A2	600	4
A3	600	10
B1	700	2
B2	700	4
B3	700	10
C1	800	2
C2	800	4
C3	800	10
D1	900	2
D2	900	4
D3	900	10

**Table 2:** Chemical constituents of rice husk ash (RHA).

Raw Materials	Constituents Oxides (wt%)					
	SiO_2_	K_2_O	CaO	Al_2_O_3_	Others	LOI
RHA	95.6 ± 0.1	1.4 ± 0.1	0.95 ± 0.1	0.71 ± 0.1	0.64 ± 0.1	0.7 ± 0.1

**Table 3:** The comparison of true density and bulk density of ZBS glass-ceramics.

Sample (s)	A	B	C	D
true density (g·cm^−3^)	4.515	4.520	4.664	4.716
bulk density (g·cm^−3^)	3.119	3.440	3.559	3.674
